# Delivering orthopaedics in Burundi: a model for humanitarian surgery in resource-limited settings

**DOI:** 10.1007/s12306-025-00934-5

**Published:** 2025-11-05

**Authors:** Giulia Cenci, Andrea Fidanza, Michele Grasso, Filippo Migliorini, Achille Contini, Francesco Falez, Manuel Giovanni Mazzoleni

**Affiliations:** 1 Santa Maria Hospital, Terni, Italy; 2 Department of Life, Health and Environmental Sciences, University of L’Aquila, L’Aquila, Italy; 3 University Hospital of Bari, Bari, Italy; 4 Department of Trauma and Reconstructive Surgery, University Hospital of Halle, Martin- Luther University Halle-Wittenberg, Halle (Saale), Germany; 5 Department of Orthopaedic and Trauma Surgery, Academic Hospital of Bolzano (SABES- ASDAA), Bolzano, Italy; 6 ASL 1 “Ospedale del Mare Hospital“, Napoli, Italy; 7 Ospedale Santo Spirito in Sassia, Rome, Italy; 8 Department of Life Sciences, Health, and Health Professions, Academic Hospital of Bolzano (SABES- ASDAA), Rome, Italy

**Keywords:** Africa, Global health, Capacity building, Musculoskeletal care, Surgical missions, Medical ethics, Low-resource environment

## Abstract

Burundi remains one of the most socioeconomically challenged countries globally, facing profound limitations in healthcare infrastructure, workforce, and access. In this context, the Italian Medical Foundation for Central Africa (FIMAC) has conducted humanitarian orthopaedic missions for over two decades in Bubanza, addressing critical musculoskeletal conditions in both paediatric and adult populations. This essay provides a comprehensive overview of the operational, clinical, and ethical dimensions of these missions. Commonly treated pathologies include chronic osteomyelitis, neglected fractures, open injuries, and congenital or acquired limb deformities—conditions frequently encountered in advanced stages due to delayed access to care. Resource-sensitive protocols guide interventions and rely heavily on collaboration with local healthcare workers, who receive targeted training in trauma management, postoperative care, and basic surgical techniques. The aim is not just to deliver urgent care but to foster sustainable improvements through capacity building and knowledge exchange. Major challenges include a lack of surgical infrastructure, limited availability of diagnostics and sterile equipment, as well as sociocultural barriers to care, such as language and traditional beliefs. Despite these constraints, the missions yield significant functional and psychosocial outcomes, particularly among paediatric patients. Ethical considerations, including informed consent, scope of practice, and cultural humility, are central to responsible practice in this setting. The personal and professional impact on participating surgeons is profound, often reshaping clinical priorities and reinforcing the humanistic foundations of the medical profession. The paper concludes by advocating for the establishment of permanent surgical facilities, structured deployments, and scalable innovations to enhance the continuity of care and address surgical inequities in low-resource settings.

## Introduction

Burundi, a small, landlocked country in Central Africa, which faced a civil war from 1993 to 2005, is among the most socioeconomically challenged nations in the world [4, 9, 10], experiencing persistent difficulties in delivering even the most basic healthcare services to its population. With a predominantly young demographic and one of the highest poverty rates globally, the country’s healthcare system is severely underdeveloped [3]. There is an acute shortage of trained medical personnel, an extremely low doctor-to-patient ratio, and widespread infrastructural inadequacies, including limited access to surgical instruments, sterilisation equipment, diagnostic imaging, antibiotics, and essential postoperative care [3]. These challenges are particularly pronounced in the field of orthopaedic surgery, which requires both technical expertise and adequate material resources, elements that are critically lacking in Burundi. In this challenging context, the Italian Medical Foundation for Central Africa (Fondazione Internazionale Medici per l’Africa Centrale, FIMAC) has conducted humanitarian orthopaedic missions for nearly 25 years in Bubanza, a small and impoverished rural town in Burundi [8]. The foundation is dedicated to providing specialised orthopaedic care to some of the most underserved populations in the country, with a particular focus on paediatric cases involving trauma, musculoskeletal infections, and congenital or acquired deformities. FIMAC’s efforts aim not only to address urgent clinical needs but also to support local healthcare systems in building long-term capacity and improving standards of care. Burundi was selected as the primary focus of FIMAC’s humanitarian engagement due to its combination of extreme poverty, fragile health infrastructure, and a disproportionate burden of orthopaedic disease. In this context, even relatively simple conditions such as fractures, dislocations, or degenerative arthritis often evolve into severe, disabling, or life-threatening complications due to delayed diagnosis, lack of early intervention, and poor follow-up capacity. These missions are driven by the dual objectives of directly treating patients and enhancing local medical capabilities through collegial cooperation and skills transfer.

Each mission team comprises exclusively highly specialised orthopaedic surgeons with substantial experience in trauma surgery, reconstructive procedures, and paediatric orthopaedics. The Burundian healthcare providers, who include anaesthesiologists, nurses, surgical assistants, and physiotherapists, collaborate closely with surgical staff throughout all stages of patient care. This partnership between international orthopaedic specialists and local multidisciplinary teams is vital to the success and sustainability of the missions. By operating within and alongside the existing healthcare infrastructure, the missions foster local engagement, procedural continuity, and mutual learning. Each mission typically lasts from two to three weeks, during which intensive clinical activity is conducted. This encompasses preoperative evaluations, surgical procedures, postoperative care, and bedside teaching. The scope of work is extensive, with a focus on conditions often seen in their most advanced forms, stemming from systemic limitations. Among these, chronic osteomyelitis, complex limb deformities, neglected fractures, and open injuries are the most frequently addressed. A central priority of the missions is the treatment of chronic osteomyelitis, a condition that is highly prevalent in Burundi. Contributing factors include inadequate management of open fractures, limited access to antibiotics, and delayed surgical care [28, 55]. The recent rise in road traffic incidents has worsened the occurrence of open fractures; however, the national health system has failed to keep up with the increasing demand for emergency orthopaedic intervention. Surgical teams employ a combination of aggressive debridement, local and systemic antibiotic therapy, and streamlined follow-up protocols to manage osteomyelitis effectively [58]. Nonetheless, the infrequency of missions and the challenges in ensuring continuity of care frequently undermine the long-term outcomes of these treatments. Congenital and acquired deformities present a significant burden in Burundi, particularly among paediatric populations. Without timely surgical intervention, these conditions often lead to lifelong disability, restricted mobility, and profound social exclusion [33, 51, 52, 62]. Corrective osteotomies and soft tissue procedures are crucial for restoring function and promoting autonomy, enabling children to participate fully in educational and social activities. These interventions not only enhance physical outcomes but also support long-term psychosocial integration and independence [24, 40–42, 61, 63]. Similarly, open fractures and trauma-related injuries represent a critical area of need. In a healthcare landscape characterised by a severe shortage of orthopaedic specialists, these injuries are often inadequately managed or left entirely untreated, thereby increasing the risk of complications such as malunion, non-union, and chronic osteomyelitis [26, 36, 62]. Timely surgical stabilisation, appropriate wound care, and adapted early mobilisation protocols are vital for preventing disability and reducing mortality. In cases where limb salvage is no longer feasible due to infection or extensive soft tissue compromise, amputation becomes a necessary and life-saving measure. However, these procedures must be accompanied by rehabilitation efforts, and when feasible, access to prosthetic devices, both of which are essential for optimising functional recovery and supporting reintegration into community life [43].

This essay provides an overview of orthopaedic missions in Burundi, placing particular emphasis on their practical, operational, and ethical dimensions. Although contextual constraints frequently limit clinical data collection, the missions offer valuable insights into delivering high-complexity surgical care in severely resource-limited settings. The article aims to share these experiences with the broader medical and humanitarian community, offering reflections that may serve as a guide and inspiration to other healthcare professionals considering similar work. This essay is organised into thematic sections that explore key challenges and strategies encountered during the missions. These include adapting to local healthcare realities, training and collaborating with local personnel, managing scarce resources, and navigating difficult ethical decisions. The final section is devoted to the personal and professional impact of these missions on participating surgeons, culminating in a set of strategic recommendations for future humanitarian initiatives. Through this contribution, we aim to highlight the crucial role of specialised surgical missions in addressing global health disparities and advocate for more structured, recurrent, and sustainable models of humanitarian orthopaedics care.

## Adapting to the local context

Burundi has followed a markedly different path of sociocultural and infrastructural development compared to European nations. According to data from the World Bank [6], the country is classified among the lowest-income nations globally. Indicators of social and economic development, institutional capacity, and healthcare infrastructure remain significantly constrained. The geographic landscape consists predominantly of rural and forested areas, with small towns scattered across challenging terrain. Only 11.6% of the population has access to electricity, and merely 24% use improved sanitation facilities. The majority of healthcare delivery is provided through rural and district-level hospitals, which are frequently under-resourced [2, 6]. Internet access remains exceptionally low, with only 11% of the population having connectivity, and many individuals lack basic telecommunication devices. Data access is limited and unaffordable for most. In this socio-economic context, where the average per capita income is around $193, technological advancements, including those in the healthcare sector, progress at a slow pace. Anonymous Data for Low income, Burundi [6]. Television ownership is rare; however, in certain public institutions, such as hospital waiting areas, televisions are available for communal use. These serve as focal points for public gatherings, highlighting the limited access to mass media and information. Healthcare technology is severely constrained. Diagnostic tools such as microbiological analysis, ultrasonography, and radiographic imaging (X-rays) are seldom available, and computed tomography (CT) scans are non-existent [16]. This significantly impedes accurate and timely diagnosis. The predominant language is Kirundi. While some individuals with formal education may have basic proficiency in French or English, language barriers are a significant challenge in clinical communication. The absence of professional medical interpreters necessitates reliance on non-verbal communication, body language, and hybrid linguistic expressions that combine local dialects, onomatopoeia, and partial elements of foreign languages. Occasionally, religious figures such as nuns or priests, especially those with international experience, serve as informal interpreters. Geographic, financial, and infrastructural barriers hinder patient access to healthcare [2, 16, 21]. Many patients live far from healthcare facilities, and transportation options are limited or non-existent. The need to engage in daily subsistence work and the inability to afford both medical care and a caregiver (locally known as gard du malade) contribute to delays in presenting clinical conditions. Additionally, traditional healers are frequently consulted due to cultural beliefs and their relatively greater accessibility. Establishing rapport with patients and local healthcare staff can initially seem challenging due to cultural, linguistic, and environmental differences. However, clinicians often find the local population to be receptive, respectful, and eager to interact with foreign medical professionals. European-trained clinicians are typically held in high esteem and regarded as authoritative medical figures. This facilitates trust-building, even in cases where patients may refuse proposed treatments. Understanding and integrating local perspectives is essential for achieving mutually agreeable clinical outcomes. Cultural practices may sometimes conflict with biomedical practices, necessitating a high degree of artistic sensitivity and professional adaptability. Beyond clinical competence, effective practice in this setting demands acceptance of a slower pace, both professionally and socially, reflecting the broader lifestyle and logistical limitations.

## Collaboration and training of local staff

The World Health Organisation (WHO) and the College of Surgeons of East, Central, and Southern Africa (COSECSA), which encompasses Ethiopia, Kenya, Tanzania, Uganda, Rwanda, Burundi, Mozambique, Malawi, Zimbabwe, and Zambia, have begun assessing emergency and essential trauma care. The current capacity to treat trauma and orthopaedic conditions is minimal, with specific areas of concern including the workforce, training, facilities, and equipment [16]. Social and cultural development and orientation in Burundi differ significantly from those in European countries and other African nations, with available resources being minimal. It remains common for patients to consult traditional healers, and currently, there are no national injury prevention programmes in place. Furthermore, there are no orthopaedic surgeons or dedicated orthopaedic departments within the healthcare system. Occasionally, small trauma teams from volunteer organisations visit the country to assist; however, they need structured local support to operate effectively. The Burundian healthcare workforce primarily comprises general practitioners, a few obstetricians, radiology technicians, general nurses, anaesthetic nurses, minor general surgeons, and healthcare assistants. On average, there is only one medical doctor for every 10,000 inhabitants [2]. Emergency medical services are scarce, and the growing incidence of injuries, road traffic accidents, and trauma-related deaths makes trauma and orthopaedic care relevant [2]. Fractures, dislocations, and wounds that reach the hospital are often left untreated or managed conservatively with inadequate or insufficient resources. Working as a foreign orthopaedic surgeon in such a fragile and under-resourced system presents critical challenges. Operating rooms frequently lack medically qualified anaesthesiologists, scrub nurses, C-arm radiography, or radiology technicians [16].

In an educational initiative aimed at developing local healthcare capacity, the goal must be to tailor training to the local environment’s constraints. Given the severe shortage of technology, emphasis should be placed on strengthening clinical judgement rather than relying solely on diagnostics. Some members of the surgical volunteering team have begun training local staff in an evidence-based approach to trauma care. They started by teaching fundamental skills such as identifying appropriate devices for outpatient care, wound management (both traumatic and surgical), and limb immobilisation. They also work to clarify which types of trauma require urgent attention. However, the development of structured treatment protocols remains a long-term objective. The introduction of simple, sustainable protocols requires targeted education and training. Keeping this in mind, they initiated a series of lessons for a selected group of nurses, focusing on the management of patients in preoperative and conservative treatment contexts. A trauma team must not only prioritise central trauma management but also ensure adequate postoperative care, considering that patients in Africa are twice as likely to die after surgery compared with the global average for postoperative death rates [12]. They have begun to explain the medical procedures performed and to teach basic bedside care, encouraging active participation and shared decision-making. Hands-on workshops, including casting, splinting, and wound care, can significantly enhance the skills of local healthcare providers and also improve patient outcomes. Fostering a culture of continuous learning and collaborative teamwork is essential for the long-term success of such initiatives.

## Managing limited resources

Historically, the global health community has not focused on surgical health care because it was thought to be prohibitively expensive and because surgery could divert resources from more cost-effective population-based interventions [19]. This is why the majority of organisations have mainly funded programmes that target infectious diseases, even though there is mounting evidence that surgical conditions have a substantial global burden and that many life-threatening emergencies and other conditions can be prevented from causing disability and premature death through relatively simple, cost-effective, and curative surgical procedures [39]. Indeed, surgical conditions will always account for a significant portion of a population’s disease burden, regardless of the efficacy of prevention strategies. This is especially true in developing countries where conservative treatment is not readily accessible, where the incidence of trauma is high, and there is a massive backlog of untreated surgical conditions brought on by domestic violence, falls, traffic accidents, burns, disasters, infections, and congenital disabilities [13]. The global health community has been increasingly inclined to recognise the function and significance of surgery as a crucial part of obstetric care. Still, it has not recognised the significance of expanding access to additional surgical treatments in low- and middle-income nations [19].

Surgery was born as emergency surgery. The mother of surgery is trauma surgery (Die Mutter der Chirurgie ist die Unfallchirurgie), as the Germans say in a lapidary way. The primordial surgical acts were the suturing of a wound, the stopping of a haemorrhage from a vessel, the removal of a foreign body from the organism, the splinting of a fractured segment, and the amputation of a limb [44]. Any ailment that necessitates suturing, incisions, excisions, manipulations, or other intrusive procedures is considered a surgical condition. However, although these manual acts were historically based on the principle of the social function of the doctor, the major obstacles to the development of surgical art were infections and the lack of anaesthesia. Since the development of antibiotics and selective anaesthetic methods, public health experts have finally dispelled the myth that surgery is overly expensive and are starting to acknowledge both the preventive value of surgery and the cost-effective viability of surgical care delivered in low-tech community hospitals [49]. For instance, a district hospital in Africa may save between $102 in surgical DALYs (disability-adjusted life years (DALYs). Antiretroviral treatment for HIV infection, on the other hand, is thought to save between $350 and $1,494 per day [38]. Surgery can therefore also help combat infectious diseases, including HIV, through male circumcision, as well as reduce poverty, as surgical problems often leave people unemployed. For instance, a survey conducted in Pakistan revealed a correlation between poverty and blindness, which is most frequently caused by cataracts [5]. Injuries are the leading cause of disability and mortality for African children who make it through the first four years of life. Approximately, 6–12% of all paediatric hospitalisations in sub-Saharan Africa are from trauma requiring surgery [7]. According to a survey of 17 surgical programmes in 13 South American, Asian, and African nations, 14% of the procedures were traumatic, and 40% of the surgeries were obstetric [17]. Despite the number of surgical procedures is rising, the majority of medical institutions in low-income nations are unable to offer even the most basic surgical treatments [39]. Only a small percentage of surgical procedures take place in lower-income nations, even if the majority of the world’s surgical illness burden falls among the world’s poorest; moreover, despite 70% of the world’s population living in poor and low-income countries, only 26% of surgeries are carried out in these nations, highlighting the startling differences in surgical care access throughout the world [29, 32, 46]. Furthermore, an estimated 234.2 million major surgical operations are carried out globally each year. Yet, only 3.5% of these procedures are conducted on the poorest third of the population, while 73.6% of all surgeries are performed on 30% of the world’s population. According to a study examining 56 WHO member states, reported surgical rates ranged from 23,369 per 100,000 people in Hungary to 148 per 100,000 persons in Ethiopia. The projected rate of major surgery in nations with healthcare spending under $100 per person is 295 operations per 100,000 annually. In contrast, the rate in nations with healthcare spending above $1,000 is 11,110 procedures per 100,000 annually [59]. There are fewer qualified local surgeons in poorer nations, which is one of the primary causes of their lower surgical rates. In high-income nations, for instance, there are five orthopaedic surgeons for every 100,000 people, whereas in sub-Saharan nations, there are fewer than one per million [48]. These statistics reveal the significant disparity that currently exists, but they also underscore the potential benefits that could be achieved if this injustice were addressed. To reduce this disparity, it would first be necessary to invest in the construction of infrastructure suitable for delivering these services to a large audience.

Hospitals must first have an operating room equipped with running water, power, and oxygen before they can perform safe and efficient surgery. However, having an operating room is just the first step; postoperative care and blood banking are essential for all institutions that have surgical programmes. Even a tiny district hospital needs a laboratory, anaesthetic machines and personnel who can use and maintain them, a blood bank on the premises, at least two operating rooms (for emergency and elective procedures), and a steady supply of energy [23]. Regretfully, these conditions are seldom satisfied in environments with limited resources. Electricity, running water, oxygen, and fuel are scarce at ten of the seventeen government civilian hospitals in Sierra Leone, according to Kingham et al. [30]. Basic supplies, such as oxygen and anaesthetics, were extremely scarce in these facilities. 40% of the hospitals had no oxygen, and 60% had an intermittent oxygen supply. Only 20% had an anaesthetic machine that functioned properly. According to research, evaluating 132 facilities across eight low- and middle-income nations, none of them reported a 100% uninterrupted supply of oxygen, power, and water [32]. There are severe deficiencies in the physical resources and infrastructure needed to deliver the most basic surgical treatment to save lives and avoid permanent impairment. Furthermore, in environments with limited resources, basic materials are frequently in short supply. According to survey data from Sierra Leone, only 50% of hospitals had sterilisers for their equipment, 30% had sufficient suction pumps, and 20% had sterile gloves on hand [25]. In Western sub-Saharan Africa, there is an estimated one operating room per 100,000 people, compared to 25 operating rooms per 100,000 people in Eastern Europe. On average, high-income areas had nearly 14 operating rooms per 100,000 residents. A survey revealed stock-outs of anaesthetic agents and linens, as well as a shortage of supplies, including large syringes, three-way stopcocks, and extension tubing [35]. Poorer countries also have fewer specialist centres. For example, in the USA, there is one heart centre per 120,000 people, in Africa, one per 33,000,000 people, and in Asia, one per 16,000,000 people [45]. Finally, people in low-income countries are unable to receive surgical care from several other infrastructure problems. For example, poor road conditions make even the shortest trips difficult, expensive, and time-consuming for the average patient [30].

The shortage of physicians and nurses in Africa has been well-documented: the continent bears over 25% of the world’s illness burden yet has just 2% of the world’s health professionals. Less than ten surgeons in Sierra Leone, for instance, are fully qualified, and only one of them is under 50 [32]. Out of 30 million people, only 75 general surgeons in Uganda are fully qualified [1]. In contrast to the USA, where there are 256 doctors per 100,000 people, Tanzania has just two doctors per 100,000 [14]. There are 21 district hospitals in Malawi, but none of them employ a permanent surgeon [34]. The lack of surgeons in underdeveloped nations is primarily caused by the lengthy training required, the high expense, and the brain drain that occurs when doctors in higher-income nations can earn more money. Not only is there a severe lack of medical professionals to perform surgery and provide anaesthesia, but the distribution of those qualified to do surgery is also unbalanced. Rural communities that are most in need of surgical treatment face exceptional access hurdles, as the majority of doctors and surgeons opt to work in metropolitan regions to maximise their career and personal prospects [47]. Given the severe shortage of qualified physicians and surgeons in low-income countries, many surgical procedures are performed by mid-level providers in a practice known as task shifting. This involves assigning some medical responsibilities to less specialised healthcare providers or even using non-physician clinicians [50]. According to a recent analysis, 25 out of 47 sub-Saharan African nations use mid-level providers. Twelve of these 25 nations had minor surgery performed by these providers, seven had major surgery (including orthopaedic operations and caesarean sections), and four had anaesthesia [31]. Task-shifting is an economical way to improve access to necessary surgical treatment. Malawi, Tanzania, Mozambique, and other nations where non-physicians have received training to treat particular surgical disorders have successfully used it. With proper surgical training and supervision, general practitioners and/or paramedical personnel may be able to complete up to 85% of procedures, according to evaluations conducted in these nations [15, 18, 37]. However, even if the survival instinct and the desire to maintain steady and long-lasting health over time contribute to the development of non-medical surgeons, the ethical aspect of treating illnesses in the world’s poorest nations must always be taken into account. Throughout the therapeutic intervention, the surgeon’s primary responsibility is to safeguard and advance the patient’s interests. The patient’s welfare must come first, regardless of how justified the surgeon’s interests may be. Above all, the remainder of the clinical application of surgical ethics is based on this premise.

While it is clear that the surgeon must prioritise the patient’s needs, this idea frequently has to be weighed against the problem of informed consent. For informed consent decisions, the surgeon must educate the patient on the nature of the disease and its related management, taking into account the cultural and religious dynamics of the target group. Unfortunately, this is not always easy, especially when a language barrier exists or in countries with a non-existent legal and jurisprudential tradition. High-income doctors are often encouraged to visit poor countries and provide much-needed surgical services. There are important ethical issues that need to be considered when surgeons travel from abroad to work in developing countries. However, they may feel satisfied in helping people who would otherwise go untreated and believe they are doing good and providing a service. Surgeons must first ensure that the level of treatment and services they provide is equivalent to what they would provide in their home country, but doctors may find themselves using expired drugs that they would not use in their home country [27]. Surgeons who visit underdeveloped nations must also be aware of their limits concerning language, culture, and the diseases they can treat. Surgeons who travel overseas frequently encounter sophisticated disorders that they have never seen before. Since Hippocrates’ day, *primum non nocere* (first, do not harm “) has been the first precept of medicine. When faced with unknown pathology and without the standard surgical support mechanisms they are used to, novice surgeons who have visited exotic locations are prone to making poor, even disastrous, clinical judgements. It is imperative to avoid the inclination to prioritise ego over the patient’s best interests [57]. The moral conundrum of whom to cure is another. Even the best surgeon might find themselves in serious difficulties when the blood bank is empty or the critical care unit lacks working ventilators. Thus, both local and visiting surgeons must be careful only to take on cases that they will have the resources to treat adequately [27]. The paternalistic mentality that surgical missions embody, as well as their unintended impact of weakening local physicians and healthcare infrastructure, is additional ethical considerations. When it comes to caring for people in their own communities, local physicians must be considered the best. Therefore, surgeons travelling abroad should be aware that their actions may not ultimately benefit the very people they are trying to help; in fact, they may even be counterproductive if they do not collaborate with their local colleagues.

## Ethics and difficult decisions

The term “medical mission”, originally associated with religious missionary work, is used here to describe any humanitarian medical endeavour [60]. While individuals may be motivated by various personal reasons, the core principle behind medical missions is often a response to global health inequities and the unequal distribution of healthcare resources [54].

Most medical missions, like ours in Burundi, take place in developing countries, where healthcare professionals routinely work in resource-limited settings. These environments often differ significantly from the volunteers’ usual workspaces, requiring them to adapt to new challenges. Limited access to equipment, personnel, and infrastructure may force volunteers to practice outside their standard scope of training or experience, particularly when encountering unfamiliar pathological conditions. Cultural differences further complicate communication, expectations, values, and the decision-making process in the medical field. In such contexts, establishing mutual understanding can be challenging. Language barriers and differing cultural norms frequently exacerbate these issues, impeding effective communication and leading to misunderstandings regarding diseases, their causes, and suitable treatments [54]. Even when cultural backgrounds are shared, articulating the risks and benefits of medical interventions and reaching collaborative treatment plans can be difficult. Nevertheless, patient-centred care relies on effective communication, shared understanding, and active patient engagement to ensure optimal outcomes [56]. The communities served by these missions are inherently vulnerable, often affected by socio-economic and environmental disadvantages that limit their access to care. In many cases, they are impoverished and at risk of exploitation. This raises serious ethical concerns regarding informed consent, beneficence, coercion, autonomy, and justice, particularly in the context of surgical interventions. Surgical capacity is severely limited in low-resource countries. Infrastructural challenges, such as poor road conditions, make accessing care time-consuming, costly, and physically demanding for patients. Language and cultural barriers add further complications often exacerbating misunderstandings between patients and providers and hindering the delivery of appropriate care [30].

For instance, explaining the necessity of an amputation can be particularly challenging. In some cultures, amputation carries a strong stigma and may lead to social isolation. As a result, patients and their families may resist the procedure, even when it is crucial for the patient’s health. In such circumstances, healthcare professionals must communicate with compassion, clarity, and cultural sensitivity while maintaining the patient’s dignity. Linguistic differences, differing expectations, and the lack of standard medical screening or protocols may also strain interprofessional relationships between local and international healthcare workers. Such tensions can affect collaboration and the quality of care provided. Moreover, there is a growing need to reframe medical missions within a broader context of social justice and human rights. Assistance should not be viewed solely as an act of charity, but rather as a duty of global citizenship, a recognition that those with fewer resources have a right to health and protection [22, 53]. When humanitarian aid is perceived only as charitable work, it may justify sending any available healthcare professional overseas, regardless of their qualifications or preparedness, under the assumption that patients would be “worse off” otherwise. This mindset risks reinforcing power imbalances, weakening trust in local healthcare systems, and absolving governments of their responsibility to strengthen public health infrastructure [11]. Medical volunteers may grapple with the realisation that their work addresses only the symptoms of much deeper systemic issues. The inability to enact lasting change at national or international levels can lead to frustration, especially when volunteers feel they are offering only a temporary “band-aid” solution [20]. This disillusionment may disrupt team cohesion and, more critically, influence clinical decision-making, potentially compromising patient care. Ultimately, while medical missions serve a vital role in addressing urgent healthcare needs, they must be guided by ethical principles, cultural humility, and a commitment to sustainable impact. The goal should always be to deliver the highest possible standard of care in a way that respects local systems, empowers communities, and upholds the dignity of every patient.

## Personal and professional impact

Participating in orthopaedic humanitarian missions in Burundi has resulted in significant personal and professional growth. The austere medical environment, characterised by the near-complete absence of specialised resources, forces every surgeon to rely not only on their foundational surgical skills but also on ingenuity, adaptability, and essential clinical judgement. Working without the refined tools and protocols of Western medicine revitalises the clinician’s reliance on hands, mind, and heart, often devoid of ancillary support. This return to the fundamentals of surgical care presents both a technical challenge and a rediscovery of purpose. Exposure to such conditions sharpens decision-making. The inability to rely on comprehensive diagnostic tools cultivates a heightened sense of clinical acumen, where each sign and gesture of the patient carries diagnostic significance that is frequently overlooked in more technologically equipped environments. The demands of this setting do not permit indecision: the courage to operate stems not from certainty but from the awareness that, quite often, one’s action represents the patient’s only chance. In these moments, one learns to navigate ambiguity with determination, recognising that perfection may be a luxury, but compassionate intervention is a necessity.

This experience also serves as an education in humility. The lack of infrastructure, such as adequate anaesthesia equipment, sterile surgical instruments, or even basic wound dressings, highlights the chasm between need and capacity. Operating in such a context reveals the limitations not only of technology but also of the human body and spirit when unsupported by systems. These limitations become the canvas upon which teamwork is both tested and refined. With local anaesthetists, nurses, and hospital staff forming the backbone of daily operations, successful outcomes depend entirely on mutual respect, clear yet often challenging communication, and an unwavering sense of shared purpose. Hierarchies collapse in the face of collective struggle, and each team member becomes indispensable. Emotional resilience is as vital as surgical competence. Each day begins with a silent battle: the internal tension between the desire to help and the reality of what is possible. The physical toll—pain in the legs, oppressive heat, sweat-soaked scrubs—fades behind the mental weight of knowing that one failed fixation, one missed infection, may cost a child not just a limb, but an entire life trajectory. Yet, within these hardships there lies an unexpected wellspring of inspiration. The faces of young patients, smiling and trusting, unaware of the stakes, serve as reminders of the value of the presence and doing what one can with what one has. It is in these children that one finds extraordinary resilience. Their smiles, often toothless and shy, hold a power that transcends language or explanation. Children without family, crawling through hospital corridors between the beds of the sick, embody both tragedy and tenacity. Their capacity to play, to hope, and to trust amid despair speaks to a level of human strength that few environments in the developed world can reveal. These are not merely patients; they are teachers. They offer lessons in courage, in dignity without demand, in survival through simplicity. Each mission carries with it stories of perseverance: the boy who loses a limb but sheds only a single tear upon understanding that his life has been saved; the mother who walks miles barefoot, carrying her child in hopes of care, and smiles upon departure despite a diagnosis no more optimistic than her arrival. These moments redefine the concept of healing. Sometimes, healing means restoring function. Other times, it means offering the dignity of attention and the gift of being seen. Such experiences fundamentally alter one’s perspective on global health and equity. They unmask the illusion of meritocracy in medical care and lay bare the arbitrariness of access. The moral implications of these disparities are impossible to ignore and give rise to a renewed ethical commitment, not only to continue such missions but also to advocate for systemic change. They remind us that being born into access is not an achievement, but a responsibility. Ultimately, these missions reignite the vocation of medicine. They remind us why we became doctors: to serve.

## Conclusion

Medical excellence lies not only in technical advancement but in empathy, adaptability, and meaningful action. The experience in Bubanza highlights the importance of interdisciplinary collaboration, human-centred care, and sustainable strategies, particularly through local empowerment, continuity of services, and educational exchange. Future missions should focus on establishing a permanent surgical facility, ensuring regular deployment of orthopaedic teams, and advancing context-specific innovations, such as local antibiotic carriers for osteomyelitis. These efforts redefine not only clinical practice but the deeper purpose of our profession. This report lacks generated, reported, or analysed data. Yet, in Africa, the pressing need is not just for research alone, but for tangible aid. In such settings, the sharing of lived experiences and preparing younger colleagues for the realities they will encounter may prove more formative than reading quantitative results, particularly where the findings of randomised controlled trials are limited. What is required is ingenuity, patience, sacrifice, and the fortitude to endure, even when confronted with inevitable disappointments.

## Data Availability

No datasets were generated or analysed during the current study.
